# Involvement of Histamine H_3_ Receptor Agonism in Premature Ejaculation Found by Studies in Rats

**DOI:** 10.3390/ijms23042291

**Published:** 2022-02-18

**Authors:** Kazuhiro Kiyohara, Daisuke Uta, Yuuya Nagaoka, Yurika Kino, Hideki Nonaka, Midori Ninomiya-Baba, Takuya Fujita

**Affiliations:** 1 Research Unit/Neuroscience, Sohyaku, Innovative Research Division, Mitsubishi Tanabe Pharma Corporation, Yokohama 227-0033, Japan; nagaoka.yuuya@ma.mt-pharma.co.jp (Y.N.); nonaka.hideki@me.mt-pharma.co.jp (H.N.); ninomiya.midori@mv.mt-pharma.co.jp (M.N.-B.); fujita.takuya@ms.mt-pharma.co.jp (T.F.); 2 Department of Applied Pharmacology, Faculty of Pharmaceutical Sciences, University of Toyama, Toyama 930-0194, Japan; 3 Digital Transformation Department, Mitsubishi Tanabe Pharma Corporation, Tokyo 100-8205, Japan; kino.yurika@mu.mt-pharma.co.jp

**Keywords:** histamine H_3_ receptor, H_3_R, premature ejaculation, electrophysiology, in vivo extracellular recording, copulatory behavior

## Abstract

Several of the drugs currently available for the treatment of premature ejaculation (PE) (e.g., local anesthetics or antidepressants) are associated with numerous safety concerns and exhibit weak efficacy. To date, no therapeutics for PE have been approved in the United States, highlighting the need to develop novel agents with sufficient efficacy and fewer side effects. In this study, we focused on the histamine H_3_ receptor (H_3_R) as a potential target for the treatment of PE and evaluated the effects of imetit (an H_3_R/H_4_R agonist), ciproxifan (an H_3_R antagonist), and JNJ-7777120 (an H_4_R antagonist) in vivo. Our in vivo electrophysiological experiments revealed that imetit reduced mechanical stimuli-evoked neuronal firing in anesthetized rats. This effect was inhibited by ciproxifan but not by JNJ-7777120. Subsequently, we evaluated the effect of imetit using a copulatory behavior test to assess ejaculation latency (EL) in rats. Imetit prolonged EL, although this effect was inhibited by ciproxifan. These findings indicate that H_3_R stimulation suppresses mechanical stimuli-evoked neuronal firing in the spinal–penile neurotransmission system, thereby resulting in prolonged EL. To our knowledge, this is the first report to describe the relationship between H_3_R and PE. Thus, H_3_R agonists may represent a novel treatment option for PE.

## 1. Introduction

According to the International Society for Sexual Medicine, men with lifelong and acquired premature ejaculation (PE) evidently share the dimensions of short ejaculatory latency (EL), reduced or absent perceived ejaculatory control, and negative personal consequences [1,2,3]. According to their guidelines, pharmacological, psychological/behavioral, educational, and combination treatment interventions may be appropriate for PE [1]. Researchers have confirmed the efficacy of some drugs for PE, including selective serotonin reuptake inhibitors (SSRIs), antidepressants, and local anesthetics applied to the glans penis. However, local anesthetics can cause local allergic reactions in patients and their partners following application to the penis, in addition to decreasing satisfaction during sexual intercourse through the diffusion of drugs [3]. Moreover, SSRIs and antidepressants can cause a high frequency of adverse effects, such as dizziness and nausea, which is inconvenient and associated with safety concerns [3,4]. To date, several drugs have been used off-label. Dapoxetine and local anesthetics have been approved in several countries within Europe. On the other hand, no pharmacological treatments have been approved for PE by the Food and Drug Administration in the United States [5,6]. These issues necessitate the development of highly convenient novel therapeutics with sufficient efficacy and fewer adverse effects [7].

In this study, we focused on the histamine H_3_ receptor (H_3_R) as a potential target for the treatment of PE. H_3_R is an inhibitory G protein-coupled receptor that functions as an autoreceptor of histamine but also affects neurotransmitter release [8,9]. It is principally expressed by the neuronal cells of the central nervous system; however, its expression on peripheral nerves, particularly the Aβ sensory nerves, has been reported in several studies [8,10,11]. The activation of H_3_R in peripheral sensory neurons inhibits mechanical stimulation-induced pain [10,11]. Thus, H_3_R stimulation can suppress the neural activity evoked by mechanical stimulation in the penis. In the context of male sexual function, histamine relaxes the smooth muscle of the corpora cavernosa and may induce erection via histamine H_1_ receptors (H_1_Rs), histamine H_2_ receptors (H_2_Rs), and H_3_Rs [12,13,14]. The use of H_2_R antagonists has been observed to decrease erectile function [14]. However, the role of H_3_R in ejaculation function has not been investigated, and its mechanism of action remains unknown.

In this study, we aimed to evaluate the potential of H_3_R as a therapeutic target for PE. To achieve this aim, we conducted electrophysiological and behavioral experiments using imetit (an H_3_R/H_4_R agonist [15,16]), ciproxifan (an H_3_R antagonist [17]), and JNJ-7777120 (an H_4_R antagonist [18]) to characterize the role of H_3_R in ejaculation prolongation.

## 2. Results

### 2.1. In Vivo Electrophysiological Recording from the Pelvic Nerve

#### 2.1.1. Effects of Imetit on Mechanical Stimuli-Evoked Firing at the Peripheral Nerve

**Figure 1:**
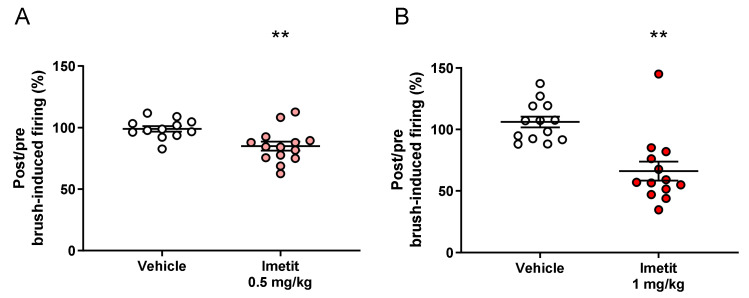
Imetit suppresses penile mechanical stimuli-evoked firing of the peripheral nerve: (**A**) 0.5 mg/kg and (**B**) 1.0 mg/kg. The genital area was first stimulated with a brush, following which evoked firing was evaluated. The post and pre-administration ratios are displayed. Data are presented as the mean ± S.E.M. ** *p* < 0.01 vs. vehicle group analyzed using Student’s *t*-test (n = 12–14).

In our electrophysiological experiments, we investigated the effect of imetit on mechanical stimuli-evoked firing of the pelvic nerve before and 5 min after the administration of the test compound. The firing changes evoked by brush stimulation in the genital area were 99% and 85% in the vehicle and imetit (0.5 mg/kg) groups, respectively (Figure 1A). The firing changes evoked by brush stimulation in the genital area were 106% and 66% in the vehicle and imetit (1.0 mg/kg) groups, respectively (Figure 1B). Imetit significantly inhibited peripheral nerve firing evoked by brush stimulation in the genital area (*p* < 0.01 vs. vehicle group analyzed by Student’s *t*-test).

#### 2.1.2. H_3_R Antagonism on the Suppressive Effect of Imetit on Mechanical Stimuli-Evoked Firing at the Peripheral Nerve

**Figure 2:**
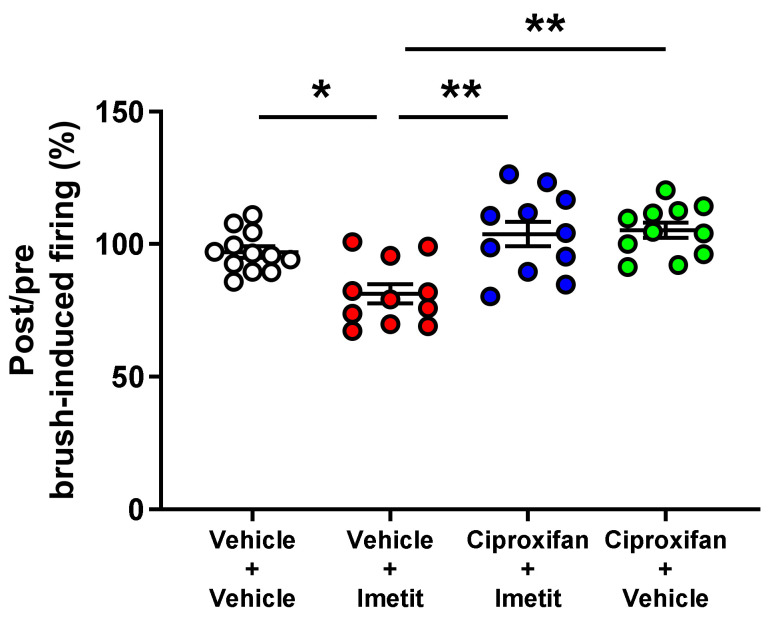
Ciproxifan (H_3_R antagonist) inhibits the suppressive effect of imetit on neuronal firing of the peripheral nerve. The genital area was first stimulated with a brush, following which evoked firing was evaluated. The post and pre-administration ratios are displayed. Data are presented as the mean ± S.E.M. * *p* < 0.05 and ** *p* < 0.01 analyzed using Tukey’s multiple comparison test (n = 11–12).

We examined the antagonistic effect of ciproxifan on the suppressive effect of imetit on mechanical stimuli-evoked firing of the pelvic nerve (also as described in Section 2.1.1). As shown in Figure 2, the firing changes evoked by brush stimulation in the genital area were 97%, 81%, 104%, and 105% in the vehicle–vehicle group, vehicle–imetit group, ciproxifan–imetit group, and ciproxifan–vehicle group, respectively. Thus, imetit (1 mg/kg) significantly inhibited peripheral nerve firing evoked by brush stimulation in the genital area (*p* < 0.05, vs. vehicle–vehicle group analyzed by Tukey’s multiple comparison test). In contrast, ciproxifan (1 mg/kg) antagonized the inhibitory effect of imetit on peripheral nerve firing evoked by brush stimulation in the genital area (*p* < 0.01 vs. vehicle–imetit group analyzed by Tukey’s multiple comparison test).

### 2.2. In Vivo Extracellular Recording of Neurons in the Deep Dorsal Horn

#### 2.2.1. Effects of Imetit on Mechanical Stimuli-Evoked Firing of the Spinal Dorsal Horn

**Figure 3:**
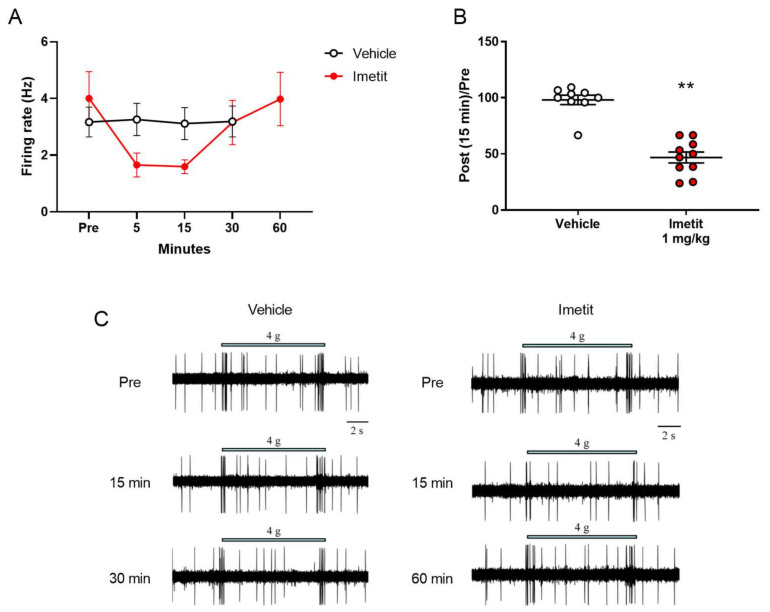
Imetit (1.0 mg/kg) suppressed penile mechanical stimuli-evoked firing of the spinal dorsal horn. The genital area was stimulated with a von Frey filament (vFF), following which we evaluated evoked firing: (**A**) temporal changes in the firing rate, (**B**) post and pre-administration ratios, (**C**) representative trace of vFF-evoked firing recorded from the vehicle or imetit group. Data are presented as the mean ± S.E.M. ** *p* < 0.01 vs. vehicle group analyzed using Student’s *t*-test (n = 9–10).

We measured the temporal changes in mechanical stimuli-evoked firing using a von Frey filament (vFF; 4.0 g), as well as the effect of imetit on firing, of the spinal dorsal horn before and after the administration of the test compound. We observed the maximal inhibitory effect of imetit on vFF stimuli-evoked firing at 5–15 min, which was recovered at 60 min (Figure 3A). Figure 3B depicts the data for the typical inhibition observed at 15 min following administration (98% in the vehicle group and 47% in the imetit group). We observed a significant effect of imetit at 15 min post administration.

**Figure 4:**
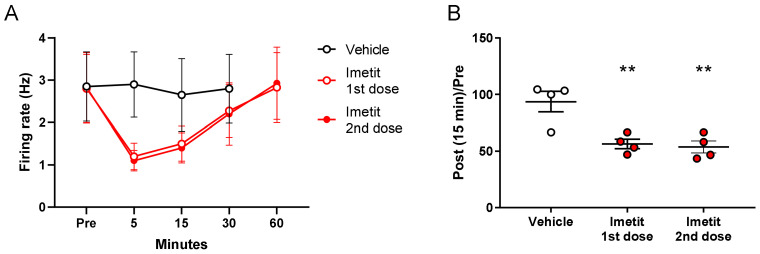
An electrophysiological system was used to perform two consecutive assessments with a 2 h interval. Imetit (1.0 mg/kg) significantly suppressed mechanical stimuli-evoked firing at the first and second doses: (**A**) temporal changes in the firing rate and (**B**) the post and pre-administration ratios. Data are presented as the mean ± S.E.M. ** *p* < 0.01 vs. vehicle group analyzed using Dunnett’s multiple comparison test (n = 4).

We also analyzed the effect of repeated imetit administration. The first administration of imetit (1 mg/kg) inhibited mechanical stimuli-evoked firing. Imetit was then re-administered 2 h after the initial administration, and an inhibition of mechanical stimuli-evoked firing (similar to the first administration) was also observed (Figure 4A). Therefore, the response to imetit was reversible under these experimental conditions, and there was no attenuation of the effect owing to repeated administration. Figure 4B presents the typical inhibition data observed at 15 min post administration (94% in the vehicle group, 56% for the first dose in the imetit group, and 54% for the second dose in the imetit group). We observed a significant effect of imetit 15 min following the first and second doses.

#### 2.2.2. Dose-Dependent Effects of Imetit on Mechanical Stimuli-Evoked Firing of the Spinal Dorsal Horn

**Figure 5:**
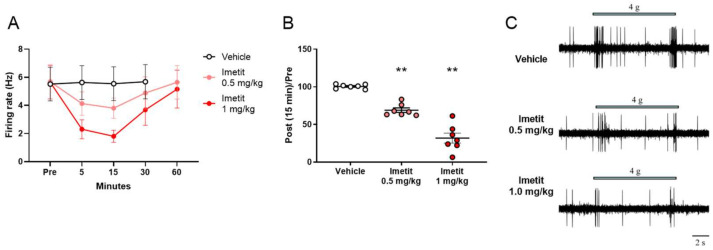
Dose-dependent effects of imetit on mechanical stimuli-evoked firing of the spinal dorsal horn: (**A**) temporal changes in the firing rate, (**B**) post and pre-administration ratios, and (**C**) representative trace of vFF-evoked firing recorded 15 min following administration in the vehicle and imetit groups. Data are presented as the mean ± S.E.M. ** *p* < 0.01 vs. vehicle group analyzed using Dunnett’s multiple comparison test (n = 7).

We investigated the dose-dependent effect of imetit on vFF stimuli-evoked firing of the spinal dorsal horn, as described in Section 2.2.1. The intravenous administration of imetit at 0.5 mg/kg and 1.0 mg/kg inhibited penile stimulus-evoked firing in a dose-dependent manner (Figure 5A). Figure 5B presents data representative of typical inhibition observed at 15 min post administration (100% in the vehicle group, 69% in the 0.5 mg/kg imetit group, and 32% in the 1.0 mg/kg imetit group). We observed a significant effect of imetit 15 min post administration.

#### 2.2.3. H_3_R and H_4_R Antagonism on the Suppressive Effects of Imetit on Mechanical Stimuli-Evoked Firing of the Spinal Dorsal Horn

**Figure 6:**
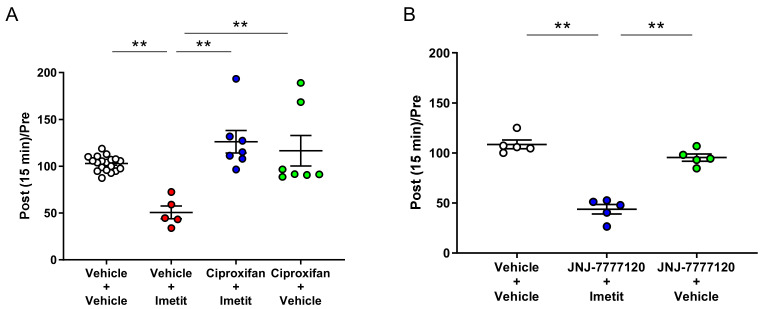
Ciproxifan (an H_3_R antagonist (**A**)), but not JNJ-7777120 (an H_4_R antagonist (**B**)), significantly inhibited the suppressive effect of imetit on neuronal firing of the spinal dorsal horn. The post and pre-administration ratios are displayed. Data are presented as the mean ± S.E.M. ** *p* < 0.01 analyzed using Tukey’s multiple comparison test (n = 5–19).

We examined the antagonistic effects of ciproxifan, an H_3_R antagonist, on the inhibitory effect of imetit on vFF stimuli-evoked firing of the spinal dorsal horn. The administration of ciproxifan prior to imetit completely reversed the inhibitory effect of imetit on vFF stimuli-evoked firing (Figure 6A; 103% in the vehicle–vehicle group, 51% in the vehicle–imetit group, 126% in the ciproxifan–imetit group, and 117% in the ciproxifan–vehicle group).

As imetit acts not only on H_3_R but also on H_4_R [15,16], we also investigated the antagonistic effect of H_4_R on the inhibitory effect of imetit on vFF stimuli-evoked firing of the spinal dorsal horn. Pre-treatment with the H_4_R antagonist JNJ-7777120 did not reverse the effects of imetit (Figure 6B; 109% in the vehicle–vehicle group, 44% in the JNJ-7777120–imetit group, and 95% in the JNJ-7777120–vehicle group). Therefore, the inhibitory effect of imetit on mechanical stimuli-evoked firing was attributed to H_3_R activation.

### 2.3. Copulatory Behavior

#### 2.3.1. Effects of Imetit on Ejaculation in Copulatory Behavior

**Figure 7:**
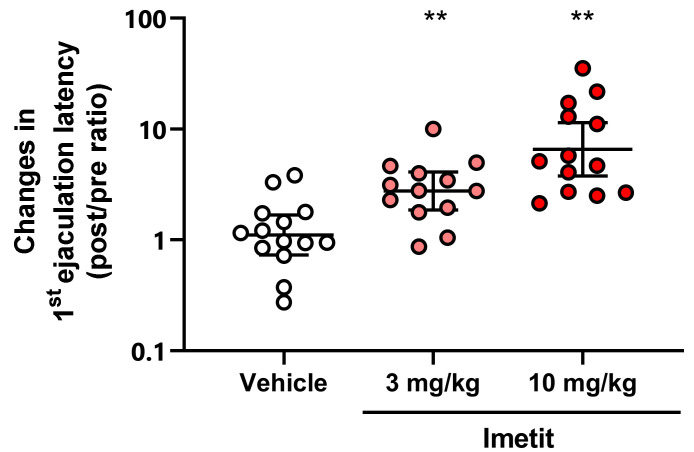
Effect of imetit on the fold change in ejaculation latency in rats. Data are presented as the geometric mean with the 95% confidence interval (n = 13). ** *p* < 0.01 vs. vehicle group analyzed using Dunnett’s multiple comparison test.

We investigated the effects of imetit on prolonged ejaculation latency (EL) in a copulatory behavior test using Wistar–Imamichi rats [19,20]. The mean baseline EL values of these rats were 59.6 s, 66.5 s, and 55.1 s in the vehicle, imetit (3 mg/kg), and imetit (10 mg/kg) groups, respectively. We performed copulatory behavior tests at 60 min following the oral administration of the vehicle or imetit. The ratio of the post-dose values to the baseline values (post/pre ratio) was calculated to assess the prolongation of EL. Imetit administered at 3 mg/kg and 10 mg/kg prolonged the EL by 3.36-fold and 6.55-fold, respectively (Figure 7). These effects were significant when compared to those observed in the vehicle group (*p* < 0.01, analyzed using Dunnett’s multiple comparison test, n = 13).

#### 2.3.2. H_3_R Antagonism on the Effect of Imetit to Prolong Ejaculation Latency

**Figure 8:**
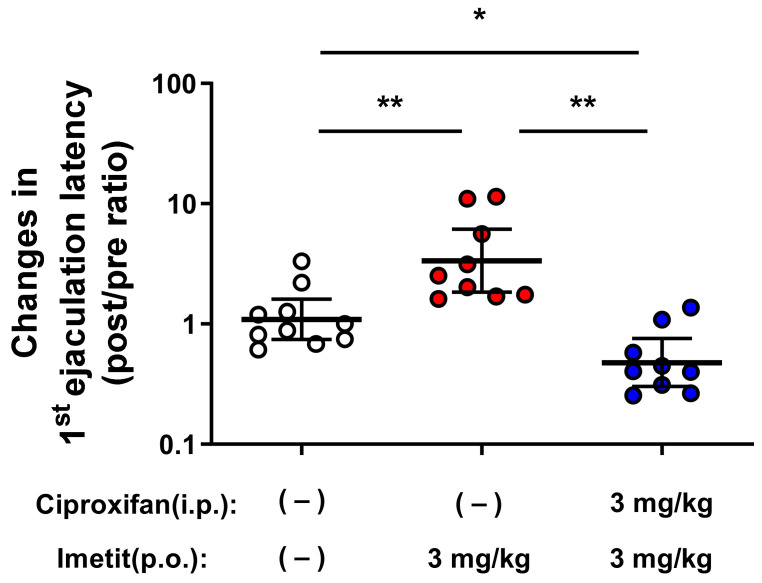
Ciproxifan (H_3_R antagonist) significantly inhibited the prolongation effect of imetit to ejaculation latency. Data are presented as the geometric mean with the 95% confidence interval (n = 9–10). * *p* < 0.05 and ** *p* < 0.01 analyzed using Tukey’s multiple comparison test.

To further characterize the role of H_3_Rs, we investigated the antagonistic effect of ciproxifan on the effect of imetit to prolong ejaculation latency. The mean baseline EL values were 77.7 s, 77.4 s, and 77.6 s in the vehicle, imetit, and combination (imetit and ciproxifan) groups, respectively. We orally administered the vehicle or imetit (3 mg/kg) 10 min following an intraperitoneal injection of the vehicle or ciproxifan (3 mg/kg) and performed copulatory behavior tests after 50 min. The post/pre ratios, indicative of the ejaculation-prolonging effect, were 1.10, 3.36, and 0.48 in the vehicle, imetit, and combination (imetit and ciproxifan) groups, respectively (Figure 8). The imetit group exhibited significantly prolonged EL when compared with the vehicle group (*p* < 0.01, vehicle group vs. imetit group analyzed by Tukey’s multiple comparison test). This effect was significantly inhibited by the combination of ciproxifan and imetit (*p* < 0.01, imetit group vs. combination group analyzed using Tukey’s multiple comparison test).

## 3. Discussion

Researchers have not yet established the mechanism underlying the contribution of H_3_R to ejaculation function. Therefore, in this study, we conducted electrophysiological and behavioral experiments to characterize the role of H_3_Rs in ejaculation function using imetit, an H_3_R agonist.

Our in vivo electrophysiological findings indicated that imetit attenuated mechanical stimuli-evoked firing of the peripheral nerve and spinal cord. Imetit is also a known H_4_R agonist [15,16]. Therefore, we examined whether H_3_ and H_4_ receptor antagonists inhibited the effects of imetit. Pre-treatment with ciproxifan, a selective H_3_R antagonist [17], inhibited the suppressive effects of imetit on neuronal firing; however, these effects were not inhibited by JNJ-7777120, a selective H_4_R antagonist [18]. We used Wistar–Imamichi rats in the in vivo copulatory behavior test, which have a shorter ejaculation latency (EL) than other general strains of rats [19,20,21,22,23]. The evaluation of EL prolongation using the aforementioned rat model may facilitate the development of drugs for the treatment of premature ejaculation (PE). Our in vivo copulatory behavior experiment revealed that imetit prolonged EL relative to the baseline, although this effect was completely inhibited by ciproxifan.

Male ejaculation results from a complex coordination between neuronal, neurochemical, and hormonal signals, including some parts of the male reproductive tract (e.g., penis, prostate, epididymis, and testis) [24]. Nonetheless, the peripheral nervous system plays an important role in the process. Stimulation from the genital area, including the penis, gets transmitted from the penis to the central nervous system via the sensory afferent nerve [24]. In addition, the spinal–penile network integrates and processes sensory stimulation [24]. Penile sensitivity is significantly greater in men with PE than in those without PE, which can be partially attributed to afferent sensory nerve function and the hyper-reflexivity of the ejaculation reflex arc [25,26]. Of the three different types of afferent sensory nerves, the Aβ fibers (skin motion, vibration, fine tactile, skin stretch, and pain) and C fibers (touch, pain) predominate in the penis [27]. Local treatment with topical anesthetics represents a candidate strategy that targets decreased sensitivity of the glans penis; however, the use of such agents is limited due to decreased sensitivity and potential effects on the partner [3]. Thus, identifying alternative strategies for reducing sensitivity remains an important concern.

H_3_Rs are inhibitory G protein-coupled receptors that function as histamine autoreceptors, although they are also known to affect neurotransmitter release [8,28,29]. H_3_Rs are predominantly expressed by the neuronal cells of the central and peripheral nervous systems [11], which are located on Aβ sensory nerves but not on C fibers [10,11]. H_3_R activation results in a series of cellular changes (e.g., reduced depolarization-induced Ca^2+^ entry into the cell, reduced neurotransmitter release, and activated G protein-gated inwardly rectifying K^+^ channels) that reduces cellular signaling [8,28,29]. In vivo, H_3_R activation suppresses mechanically induced pain behavior, even in peripheral sensory nerves [11,30]. However, the role of H_3_R in ejaculation remains unknown.

In this study, we utilized a novel in vivo extracellular recording system to investigate the effect of imetit on penile stimulation-evoked firing. Nerve firing evoked by mechanical stimulation of penile tissues was measured at the dorsal horn of the lumbosacral spinal cord (L6-S1) entering via the peripheral nerves [31,32,33]. The unmyelinated C and thinly myelinated Aδ fibers carry noxious information from the periphery to the central level, whereas Aβ fibers principally transmit tactile information. Moreover, laminae III and IV neurons are the major recipient laminae for tactile information [34]. Among the deep dorsal horn neurons, imetit decreased the frequency of vFF-evoked firing in Aβ neurons. Therefore, imetit suppressed touch-evoked firing inputs to the deep dorsal horn neurons by blocking action potential conduction through Aβ fibers. Taken together, these findings indicate that H_3_R agonists blocked nerve firing principally from Aβ fibers originating from the penis, and that H_3_ receptor agonism may represent a therapeutic agent for inhibiting subtle stimuli, such as touch sensation on the penis.

In summary, our data indicated that the intravenous administration of imetit inhibited penile stimulation-evoked firing in a reversible and dose-dependent manner at the peripheral nerves and spinal dorsal horn. Moreover, the aforementioned effect was mediated by H_3_R. Imetit treatment resulted in potent prolongation of EL in the in vivo copulatory behavior tests when administered at doses similar to those used in the electrophysiological tests. This effect was completely antagonized by ciproxifan. Hence, the stimulation of H_3_R suppressed mechanical stimuli-evoked neuronal firing in the spinal–penile neurotransmission system, thereby prolonging EL. Based on these findings, H_3_R agonists are expected to be a novel treatment for PE.

## 4. Materials and Methods

### 4.1. Animals

Seven-week-old Wistar rats (Japan SLC, Inc., Hamamatsu, Japan) were used for the electrophysiological tests (recording from the spinal dorsal horn), which was conducted at Toyama University. For the electrophysiological tests (recording from the peripheral nerve) and the copulatory behavior tests at conducted at Mitsubishi Tanabe Pharma Corporation (MTPC), 7- to 8-week-old and 10- to 17-week-old Wistar–Imamichi rats (Institute for Animal Reproduction, Ibaraki, Japan) were used, respectively.

The Wistar rats were housed under environmentally controlled conditions with a 12-h light/dark cycle (lights on at 7:00 AM), temperature (permissive range) of 23 °C (20–26 °C), and humidity (permissive range) of 55% (30–60%), with access to food and water ad libitum. The Wistar–Imamichi rats were housed under environmentally controlled conditions with a 12-h light/dark cycle (lights on at 7:00 AM and 1:00 AM for the electrophysiological tests and copulatory behavior tests, respectively), temperature (permissive range) of 23 °C (20–26 °C), and humidity (permissive range) of 55% (30–70%), with access to food and water ad libitum.

All in vivo experimental procedures were performed in accordance with the “Guiding Principles for the Care and Use of Animals in the Field of Physiological Sciences” of the Physiological Society of Japan and were approved by the Institutional Animal Care and Use Committee of Research Laboratories at the University of Toyama and MTPC. All efforts were made to minimize animal suffering and the number of animals used for the study.

### 4.2. Test Compounds

We purchased imetit dihydrobromide, ciproxifan maleate, and JNJ-7777120 from Sigma-Aldrich, Inc. (St. Louis, MO, USA). Imetit was dissolved in saline and 0.5% methylcellulose (Shin-Etsu Chemical, Tokyo, Japan) solution for the electrophysiological tests and copulatory behavior tests, respectively. Ciproxifan was dissolved in saline for both electrophysiological and copulatory behavior studies. JNJ-7777120 was dissolved in 20% (2-hydroxypropyl)-β-cyclodextrin (Sigma-Aldrich, Inc., St. Louis, MO, USA) solution for the electrophysiological tests.

### 4.3. In Vivo Electrophysiological Recording from the Pelvic Nerve

All rats were anesthetized with urethane anesthesia (1.6 g/kg, i.p.). Following their fixation in the supine position, we created a midline incision on the skin and muscle from the lower abdomen to below the diaphragm. The surrounding connective tissue was dissected to expose the pelvic nerves. We placed the pelvic nerve on a silver–silver chloride bipolar electrode connected to a bioelectric amplifier (DAM 80, World Precision Instruments, Sarasota, FL, USA) in an electric shield, following which neuronal firing activity was recorded. Micro1401-3 (Cambridge Electronic Design, Cambridge, England) was used as the interface. A catheter (PE50, Becton Dickinson, Franklin Lakes, NJ, USA) was inserted into the femoral vein to administer the test compound. Evoked firing was defined as that evoked by brush stimulation of the genital area around the penis and was determined by averaging two or more responses to 10 s of stimulation. Data analysis was performed using Spike 2 software (Cambridge Electronic Design, Cambridge, UK).

Imetit and ciproxifan were used as the test compounds and were administered intravenously at 1 mL/kg through a catheter inserted in the femoral vein. We evaluated brush-evoked firing before and 5 min following the administration of the test compounds.

### 4.4. In Vivo Extracellular Recording from the Deep Dorsal Horn Neurons

Novel methods for in vivo extracellular recording of the deep spinal dorsal horn neurons were developed by modifying previously reported methods [35,36,37,38]. Briefly, the rats were anesthetized with urethane (1.2–1.5 g/kg, i.p.), followed by thoracolumbar laminectomy, which exposed the levels from lumbar 3 (L3) to sacral 2 (S2). These rats were then placed in a stereotaxic apparatus. We excised the dura and arachnoid membranes to create an access point for the patch electrode. The surface of the spinal cord was irrigated with 95% O_2_–5% CO_2_-equilibrated Krebs solution (10–15 mL/min) at 37 ± 1 °C. In vivo extracellular recordings were generated from L6–S1 (lumbosacral region), which receives input from the penile region (lower abdomen). We performed single-unit extracellular recordings of the spinal dorsal horn (principally lamina III and IV) neurons and selected the spikes for amplitude discrimination [35,39,40]. A tungsten microelectrode (tip diameter: 25 μm, tip impedance: 9–12 MΩ) was inserted into the spinal cord of the ipsilateral side at an angle of 20–30° (latero-medial), and recordings were obtained from neurons 180 to 400 μm below the surface, which corresponded to laminae III and IV. The unit signals were amplified (EX1; Dagan Corporation, Minneapolis, MN, USA), digitized (Digidata 1400A, Molecular Devices, Union City, CA, USA), and displayed online using a special software package (Clampfit version 10.2; Molecular Devices, Union City, CA, USA). We explored a region on the skin where a touch with a cotton wisp and/or light brush produced a neural response. Mechanical stimulation was applied to the penile region for 10 s using a vFF.

Imetit, ciproxifan, and JNJ-7777120 were used as the test compounds. Imetit and ciproxifan were administered intravenously at 1 mL/kg through a catheter inserted in the femoral vein. JNJ-7777120 was administered subcutaneously at 1.5 mL/kg.

### 4.5. Copulatory Behavior Test

#### 4.5.1. Procedure

We assessed copulatory behavior in a dark room during the dark phase. Male rats were placed in observation cages (170 × 400 × 320 mm, ZKG-TMT 450, Terauchi Kagaku, Tokyo, Japan) with a wire mesh floor (KG-BED 17, Terauchi Kagaku) and habituated for more than 10 min. Following habituation, female rats in the proestrus period of their estral cycle were placed in the cage for more than 1 h to allow mating.

Before the evaluation of pharmacological efficacy, the male rats were mated six times weekly for training. Each mating was performed using a different female rat. Following the sixth mating, male rats with ejaculation latency (EL) < 150 s in the sixth test were selected for the pharmacological experiment. In addition, the EL recorded during the sixth mating was used as the baseline. Selected rats were also divided into groups to ensure uniform baseline values. From the seventh test, we performed pharmacological tests once a week to evaluate the effect of compounds on EL, each time with a different female rat. Male rats were treated with the test compounds or vehicle.

Imetit was orally administered 1 h before the onset of mating. In contrast, both ciproxifan (i.p.) and imetit (p.o.) were administered 60 min and 50 min before mating, respectively, to evaluate their combined effect.

#### 4.5.2. Observation

The first 30 min of mating was recorded using a video camera (HC-VX 985M, Panasonic, Osaka, Japan) in night mode (infrared light). One hour after the commencement of mating, the male rats were quickly returned to their home cages. We measured both EL and the fold change in EL to evaluate copulatory behavior. EL denotes the time from the first intromission to ejaculation for the first ejaculatory series. The fold change in EL (post-/pre-ratio) denotes the ratio of the baseline EL to the EL following compound administration. The cut-off value for EL was set to 30 min for male rats displaying one or more intromissions and no ejaculation during the 30 min copulatory behavior session.

### 4.6. Statistical Analysis

Data for electrophysiological tests are presented as the mean ± standard error of the mean (SEM). The average number of firings evoked by mechanical stimuli before the administration of the test compound was set to 100% and compared with changes in the number of firings post administration. For statistical analyses, we performed one- or two-way analysis of variance (ANOVA), followed by Student’s *t*-test, Dunnett’s multiple comparison test, or Tukey’s multiple comparison test. All statistical analyses were performed using SAS software (version 9.4, SAS Institute, Inc., Cary, NC, USA) using a two-tailed test with a significance level of 0.05.

In the copulatory behavior test, we calculated the geometric mean and 95% confidence interval of the fold change in EL (post/pre ratio). Statistical analyses were conducted following the logarithmic transformation of the raw value of each post/pre ratio. Moreover, we performed one-way ANOVA, followed by Dunnett’s multiple comparison test or Tukey’s multiple comparison test. All statistical analyses were performed using SAS software (version 9.4, SAS Institute, Inc., Cary, NC, USA) using a two-tailed test with a significance level of 0.05.

## 5. Conclusions

Overall, our findings indicated that H_3_Rs are selectively involved in nerve firing evoked by mechanical stimulation of the genital area. Furthermore, H_3_R stimulation acts on the peripheral nerves, including those in the penis or synaptic terminals of the spinal dorsal horn, thereby suppressing the generation of receptor potentials, action potentials, and neurotransmitter release. This inhibits signal transmission and suppresses the input of penile stimulation to the spinal cord, thus resulting in EL prolongation.

However, H_3_Rs are also expressed in the brain. Although no central side effects were observed in our pharmacological experiments, the risk of developing central side effects cannot be ruled out. Further studies are required to develop novel peripherally acting compounds to demonstrate the utility of H_3_R agonists as potent and less adverse drugs for PE treatment.

## Data Availability

The original contributions presented in the study are included in the article, and further inquiries can be directed to the corresponding author.
